# The Predictors of Obesity Hypoventilation Syndrome in Obstructive Sleep Apnea

**DOI:** 10.4274/balkanmedj.2015.1797

**Published:** 2017-01-05

**Authors:** Aylin Pıhtılı, Züleyha Bingöl, Esen Kıyan

**Affiliations:** 1 Department of Pulmonary Medicine, İstanbul Haydarpaşa Numune Training and Research Hospital, İstanbul, Turkey; 2 İstanbul University School of Medicine, Department of Pulmonary Medicine, İstanbul, Turkey

**Keywords:** Apnea hypopnea index, hypercapnia, obesity, obesity hypoventilation syndrome, obstructive sleep apnea

## Abstract

**Background:**

As obesity increases, the frequency of obstructive sleep apnea and obesity hypoventilation syndrome increases also. However, obesity hypoventilation syndrome frequency is not known, as capnography and arterial blood gas analysis are not routinely performed in sleep laboratories.

**Aims:**

To investigate the frequency and predictors of obesity hypoventilation syndrome in obese subjects.

**Study Design:**

Retrospective clinical study.

**Methods:**

Obese subjects who had arterial blood gas analysis admitted to the sleep laboratory and polysomnography were retrospectively analyzed. Subjects with restrictive (except obesity) and obstructive pulmonary pathologies were excluded. Demographics, Epworth-Sleepiness-Scale scores, polysomnographic data, arterial blood gas analysis, and spirometric measurements were recorded.

**Results:**

Of the 419 subjects, 45.1% had obesity hypoventilation syndrome. Apnea hypopnea index (p<0.001), oxygen desaturation index (p<0.001) and sleep time with SpO_2_<90% (p<0.001) were statistically higher in subjects with obesity hypoventilation syndrome compared to subjects with eucapnic obstructive sleep apnea. The nocturnal mean SpO_2_ (p<0.001) and lowest SpO_2_ (p<0.001) were also statistically lower in subjects with obesity hypoventilation syndrome. Logistic regression analysis showed that the lowest SpO_2_, oxygen desaturation index, apnea hypopnea index and sleep time with SpO_2_ <90% were related factors for obesity hypoventilation syndrome.

**Conclusion:**

Obesity hypoventilation syndrome should be considered when oxygen desaturation index, apnea hypopnea index and sleep time with SpO_2_ <90% are high.

Obesity hypoventilation syndrome (OHS) is a diagnosis of exclusion and should be distinguished from other conditions that are commonly associated with hypercapnia (1). American Academy of Sleep Medicine (AASM) defines OHS as otherwise unexplained daytime hypercapnia [partial carbon dioxide (PaCO_2_) >45 mmHg] in obese subjects with body mass index (BMI) >30 kg/m^2^ and is associated with obstructive sleep apnea (OSA) in 90% of subjects (2). The frequency of OHS and OSA increase as BMI increases (3,4,5,6). While OSA is seen in 50-77% of subjects with BMI ≥30kg/m^2^, the frequency of OHS is between 10% and 38% in obese OSA subjects (1,7,8,9,10,11,12,13,14).

Subjects with OHS have worse prognosis, poor quality of life and higher health costs compared with subjects with eucapnic OSA (15,16). The incidence of significant co-morbidities such as congestive heart failure, atherosclerotic heart disease, cor pulmonale and pulmonary hypertension in OHS is also higher compared with eucapnic obese and non-obese subjects with OSA (7,17,18). Since arterial blood gas (ABG) analysis and nocturnal capnography are not routinely performed in sleep laboratories, the incidence of OHS is underestimated. While the prevalence of OHS in the adult population is 0.15-0.3%, in OSA subjects it is estimated to be 10-20% (7,8,10,11,12,13,17). Therefore determining the risk factors for OHS is important. The primary aim of this study was to investigate the frequency and predictors of OHS in obese subjects (BMI >30 kg/m^2^) with OSA.

## MATERIALS AND METHODS

In our sleep laboratory, polysomnography is performed by Alice 5 or Compumedics E devices; recordings are performed according to the AASM guideline (19). The AASM 2007 criteria were used for sleep staging and respiratory event scoring (20). We retrospectively screened all medical records of OSA subjects with BMI >30 kg/m^2^ between 1 January 2011 and 10 March 2014. Subjects without OSA, subjects with obstructive [forced expiratory volume 1 second (FEV_1_)/forced vital capacity (FVC)<70%] and restrictive lung diseases (except obesity), use of medication effecting sleep architecture (antihistamines, antidepressant, hypnotic agents etc.), and subjects without ABG analysis were excluded from the study. The demographics, BMI (calculated as weight height-2 ratio), spirometric measurements (ZAN 74N device), daytime ABG at room air (Radiometer ABL 5), Epworth Sleepiness Scale (ESS) scores and polysomnographic data were noted. An ESS score of ≥10 was considered to be excessive daytime sleepiness (21).

OHS is defined as a combination of obesity (BMI >30 kg/m^2^), chronic daytime hypercapnia (PaCO_2_ >45 mmHg), and sleep-disordered breathing in the absence of other known causes of hypercapnia (2).

Declaration of Helsinki was followed throughout the study. The study was approved by the Ethical Committee of İstanbul University Faculty (Ethic no: 2014/1714).

### Statistical analysis

Statistical analysis was done using the SPSS 21.0 pocket program (AIMS; İstanbul, Turkey). Continuous variables were expressed as mean and standard deviation while categorical variables were expressed as number and percentage. The concordance of normal distribution of all variables was calculated with the Shapiro-Wilk test. If the data were not normally distributed, we used nonparametric tests for dependent variables. Comparisons between groups were carried out with Mann-Whitney U test or Student’s t-test. Categorical variables were compared with the chi-square test. Correlation coefficients were calculated using Spearman correlation analysis. Logistic regression analysis was used to determine the related factors of OHS. The area under the receiver operator characteristic (ROC) curve for polysomnographic parameters was analyzed to determine a cut-off level for identifying OHS. To assess the predictive performance of polysomnographic parameters, multiple 2x2 contingency tables were used to calculate sensitivity and specificity. Statistical significance was considered as p<0.05.

## RESULTS

We screened 500 medical records retrospectively and 81 subjects were excluded because of the exclusion criteria. The study included 419 subjects with OSA (58% females, mean age: 51.8±10.9/years, 51.6% with severe OSA, 25.5% with moderate OSA and 22.9% with mild OSA). Of the 419 OSA subjects, 45.1% had OHS (n=189; 53.9% females, mean age: 52.9±10.5/years; 63% with severe OSA, 18.5% with moderate OSA, 18.5% with mild OSA). Table 1 shows the demographics and Table 2 shows the polysomnographic data of all subjects, eucapnic OSA subjects (n=230) and OHS subjects (n=189). Mean age and gender distribution were similar in groups of OHS and eucapnic OSA. Comorbidities were observed in 75.7% (n=317) of all subjects. These were hypertension (69.7%), hyperlipidemia (51%), diabetes mellitus (46.4%), ischemic heart disease (21.4%) and cerebrovascular disease (2.7%). For the 131 subjects who have all data for metabolic syndrome (MS), MS frequency was 72.5% (n=95).

When we compared the data of age and gender matched subjects with eucapnic OSA and OHS, OHS subjects had higher BMI, worse spirometric measurements and worse ABG (Table 1). Comorbidities and MS frequency were similar in both groups. The frequency of subjects with daytime SpO_2_ <95% was significantly higher in the OHS group (48.9% vs 10.4%, p<0.001). In the OHS group, AHI, oxygen desaturation index (ODI) and sleep time with SpO_2_ <90% were significantly higher and the nocturnal mean SpO_2_ and lowest SpO_2_ were significantly lower than eucapnic OSA subjects. Also, AHI ≥30/hour, ODI ≥10/hour and subjects with sleep time with SpO_2_ <90% ≥20% were significantly more frequent in the OHS group (p<0.001, p<0.001, p<0.001 respectively). A comparison of polysomnographic data of OHS and eucapnic OSA groups is shown in Table 2.

BMI was ≥40 kg/m^2^ in 69.4% (291/419) of all subjects, most of whom were female (63.6%). For the subjects with BMI ≥40 kg/m^2^, OSA severity was 53.6% severe, 22% moderate and 24.4% mild. OHS frequency was 49.5% (n=144), ODI was ≥10/hour in 99.2% (n=133/134) and sleep time with SpO_2_ <90% was ≥20% in half of OHS subjects with BMI ≥40 kg/m^2^ (n=72). Table 3 shows the demographics, ABG analysis results, polysomnographic and spirometric meausrements of OHS and eucapnic OSA subjects with BMI ≥40 kg/m^2^.

Spearman correlation showed that PaCO2 was correlated with increased age, BMI, ESS, bicarbonate (HCO_3_), AHI, ODI and sleep time with SpO_2_ <90% (r=0.099, p=0.043; r=0.283, p<0.001; r=0.158, p=0.001; r=0.640, p<0.001; r=0.269, p<0.001; r=0.414, p<0.001; r=0.488, p<0.001 respectively). On the other hand, PaCO_2_ was correlated with decreased FEV_1_%, FVC%, daytime partial oxygen saturation (PaO_2_) and nocturnal mean SpO2, as well as having the lowest SpO2 levels (r=-0.244, p<0.001; r=-0.223, p<0.001; r=-0.471, p<0.001; r=-0.431, p<0.001; r=-0.459, p<0.001 respectively).

Parameters correlating with PaCO_2_ were used for logistic regression analysis. PaO_2_ (OR=1.072, p<0.001), HCO_3_ (OR=0.568, p<0.001), ODI (OR=0.977, p=0.034) and nocturnal lowest SpO_2_ (OR=1.043, p=0.005) were found to be related factors. When logistic regression analysis was performed with only polysomnographic parameters, lowest SpO_2_, ODI, AHI, and sleep time with SpO_2_ <90% were found to be related to OHS diagnosis (OR=1.001, p=0.042; OR=0.971, p=0.001; OR=1.023, p=0.007; and OR=0.969, p=0.026 respectively). According to the ROC curves, cut-offs were determined for these OHS-related parameters. ROC was 0.632 for ODI. ODI ≥36/hour had a sensitivity of 70% and specificity of 62%. ROC was 0.706 for AHI. AHI ≥25/hour had sensitivity of 70%, specificity of 60%. ROC was 0.703 for sleep time with SpO2 <90%. Sleep time with SpO2 <90% ≥10% had a sensitivity of 65% and a specificity of 70%. In the ROC analysis, we could not find a cut-off for the lowest SpO_2_ which gives a satisfactory discrimination for OHS diagnosis. The sensitivity and specificity of oxygen saturation (SaO_2_) <95%, for identifying OHS was 49.24% and 90.11%, respectively.

## DISCUSSION

Our study is the largest study to investigate the OHS frequency and predictors in obese OSA subjects. Additionally we first demonstrated a diagnostic performance of the nocturnal oxygenation parameters such as ODI, sleep time spent with SpO_2_ <90%, and lowest SpO_2_ by giving a cut-off. In our study, 45.1% of obese OSA subjects had OHS. This frequency was higher when compared to similar studies in the literature (14,22). The highest frequency in the literature is 38% but obstructive lung disease was not an exclusion criteria in that study (22). In a study which included 150 subjects with BMI >35 kg/m^2^, the frequency of OHS was also lower (31%) than our result (3). Higher frequency of OHS in our study can be explained by 70.9% of the subjects having BMI ≥40 kg/m^2^.

Co-morbidities such as hypertension, type 2 diabetes mellitus, cardiac failure, severe pulmonary hypertension, and risk of death are more common in OHS subjects when compared to obese subjects without OSA or obese OSA subjects (3,7,23,24,25,26,27). In our study, the rate of co-morbidities was high in both eucapnic OSA and OHS groups. However, we did not find any significant difference. In our study, we recorded comorbidities according to patient statements and the use of medication. Since we did not analyze laboratory parameters for diagnosing comorbidities, our rates may be underestimated.

In the previous studies comparing OHS subjects with eucapnic OSA subjects, higher BMI and worse spirometric measurements, ABG parameters and nocturnal oxygenation were reported in subjects with OHS (8,11,12,14,27,28). In a recent study performed by Basoglu and Tasbakan (27), a comparison of 59 OHS subjects and 295 obese OSA subjects showed that OHS subjects had a higher rate of daytime sleepiness, decreased FVC, FEV_1_ and PaO_2_ and increased PaCO_2_ and HCO_3_ levels. Mean and lowest SpO_2_ during sleep were decreased, and sleep time with SpO_2_ <90% was increased in OHS (27). The same study found sensitivity and specificity of daytime SaO_2_ <95% for OHS diagnosis to be 64.4% and 73.9% respectively. Our study similarly showed decreased spirometric measurements, PaO_2_, SaO_2_ and increased PaCO_2_, HCO_3_ in OHS subjects when compared to eucapnic OSA subjects. There was a significant increase in the number of subjects with daytime saturation <95% in our OHS group when compared to the eucapnic OSA group (48.9% vs 10.4%, p<0.001). Sensitivity and specificity of SaO_2_ <95% for the diagnosis of OHS was 49.24% and 90.11%, respectively. Similar to the findings of Basoglu and Tasbakan (27), we also demonstrated that daytime HCO_3_ in ABG analysis can be a predictor for OHS. Recently, Macavei et al. (28) reported similar findings in a study of 525 subjects of which only 65.5% were obese and only 216 had BMI ≥35 kg/m^2^.

Lowest SpO_2_, ODI, AHI and sleep time with SpO_2_ <90% were related factors for OHS diagnosis in our study. As a result, we believe that nocturnal hypoxemia is the most important polysomnographic finding for OHS. Our study is the first to demonstrate ODI as a related factor for OHS. We also found that AHI was significantly higher in OHS when compared to eucapnic OSA subjects and we reported AHI as a related factor for OHS. This finding was similar to the findings of Mokhlesi et al. (8) and in contrast to the findings of Resta et al. (12) and Basoglu and Tasbakan (27).

OHS diagnosis is more common in men in the literature but the reason for this dominance is not as clear as it is in OSA (1). In our study group, there was a female dominance and OHS was more frequent in women. This is unusual for subjects with OSA. There are only three previous studies which found similar dominance of females in OHS (3,25,29).

Although retrospective study design is a limitation, our study has the largest population when compared to previous studies. Largest sample size and homogenous study population make our findings important and we believe that our study will contribute to the literature. Additionally giving a cut-off for OHS related parameters is another good point of our study. On the other hand, we did not include any non-OSA OHS subjects, which may be criticized.

In conclusion, OHS is frequently seen in obese subjects with OSA diagnosis. OHS should be considered and ABG should be analyzed in obese subjects with ODI ≥36/hour, AHI ≥25/hour and sleep time with SpO_2_ <90% ≥10%.

## Figures and Tables

**Table 1:**
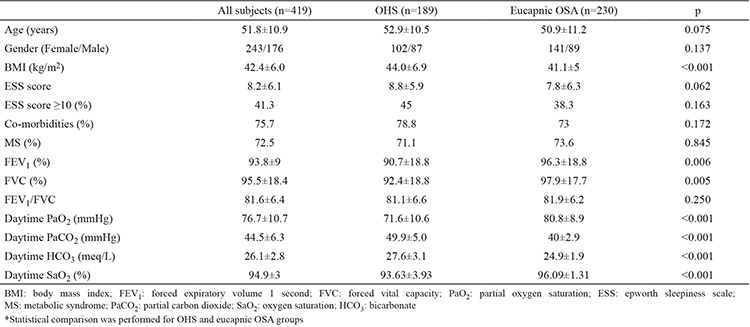
Demographics of all obese OSA subjects, OHS group and eucapnic OSA group

**Table 2:**
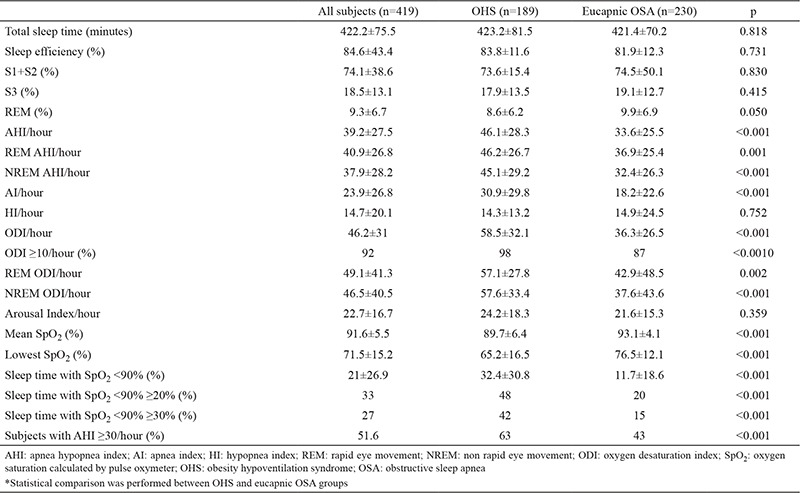
Polysomnographic parameters of all obese OSA subjects, OHS and eucapnic OSA groups

**Table 3:**
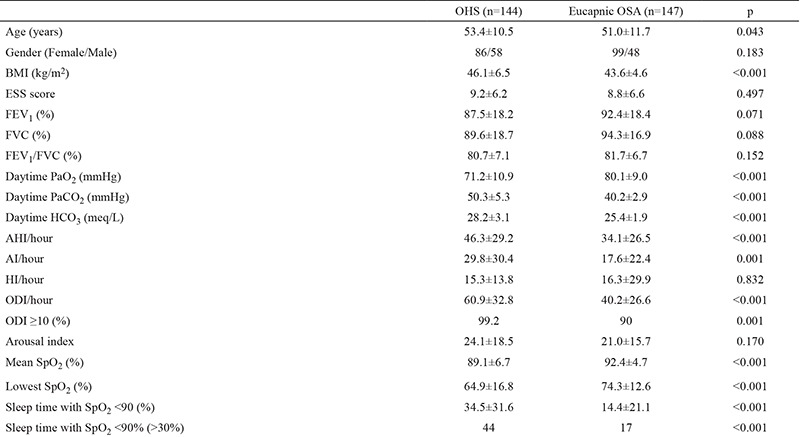
General, clinical and polysomnographic properties of subjects with BMI ≥40 kg/m^2^
